# Transcriptome profiling during a natural host-parasite interaction

**DOI:** 10.1186/s12864-015-1838-0

**Published:** 2015-08-28

**Authors:** Seanna J. McTaggart, Timothée Cézard, Jennie S. Garbutt, Phil J. Wilson, Tom J. Little

**Affiliations:** Institute of Evolutionary Biology, School of Biological Sciences, Ashworth Laboratories, University of Edinburgh, Edinburgh, EH9 3JT UK; Edinburgh Genomics, Ashworth Laboratories, University of Edinburgh, Edinburgh, EH9 3JT UK; Centre for Immunity, Infection and Evolution, School of Biological Sciences; Ashworth Laboratories, University of Edinburgh, Edinburgh, EH9 3JT UK

**Keywords:** *Daphnia magna-Pasteuria ramosa*, Innate immunity, RNA-Seq, Differential expression, Candidate genes

## Abstract

**Background:**

Infection outcome in some coevolving host-pathogens is characterised by host-pathogen genetic interactions, where particular host genotypes are susceptible only to a subset of pathogen genotypes. To identify candidate genes responsible for the infection status of the host, we exposed a *Daphnia magna* host genotype to two bacterial strains of *Pasteuria ramosa,* one of which results in infection, while the other does not. At three time points (four, eight and 12 h) post pathogen exposure, we sequenced the complete transcriptome of the hosts using RNA-Seq (Illumina).

**Results:**

We observed a rapid and transient response to pathogen treatment. Specifically, at the four-hour time point, eight genes were differentially expressed. At the eight-hour time point, a single gene was differentially expressed in the resistant combination only, and no genes were differentially expressed at the 12-h time point.

**Conclusions:**

We found that pathogen-associated transcriptional activity is greatest soon after exposure. Genome-wide resistant combinations were more likely to show upregulation of genes, while susceptible combinations were more likely to be downregulated, relative to controls. Our results also provide several novel candidate genes that may play a pivotal role in determining infection outcomes.

**Electronic supplementary material:**

The online version of this article (doi:10.1186/s12864-015-1838-0) contains supplementary material, which is available to authorized users.

## Background

The invertebrate immune response is well characterised (though not to the extent of the vertebrate system), especially in the fruit fly *Drosophila,* and in particular where the response has been stimulated by injury, the injection of a general immunoelicitor, or forced infection with a generalist or microbe. These studies have indicated that the invertebrate innate immune system can broadly distinguish between fungal, viral or bacterial invaders, and also between gram-negative and gram-positive bacteria [1].

Less is known about naturally infecting, coevolving host-pathogen systems, and in particular those characterised by genetic specificity, where the probability of infection, pathology, or parasite transmission success depends on the specific pairing of host and pathogen genotypes. For example, markedly different infection outcomes occur when different genotypes from a single population of the crustacean *Daphnia* are exposed to different strains of a single bacterial species [2] or when different genotypes of *Anopheles* are exposed to different *Plasmodium* genotypes [3]. In these cases, it is clear that the probability of infection is not a characteristic of the host genome alone. Instead, infection outcome is determined by the specific interaction between the host and pathogen genomes. This context-dependent outcome, termed genetic specificity, may be common, and so to reveal the complete molecular landscape of host–pathogen interactions we need to account for genetic specificity [4]. For example, in the bumblebee-trypanosome host-parasite system, Barribeau et al. (2014) [5] demonstrated that gene expression profiles depended on the host-parasite genotype combination, showing that genetic specificity has an underlying molecular basis.

Genomic resources now available for the crustacean *Daphnia* are increasing opportunities to elucidate the genetic basis of natural responses to a wide array of key ecological parameters, including responses to toxins, predators and pathogens. The species *D. pulex* was the first sequenced crustacean, and while not a model for host parasite interactions, the availability of this species’ genome has aided the preparation and annotation of the genome sequence of *D. magna,* which is a model for the study of coevolution. Indeed, the parasitic interaction between *D. magna* and its naturally coevolving bacterium *Pasteuria ramosa* offers key examples of both genetic specificity [2, 6] and frequency-dependent coevolution through time [7]. Here, we present the complete transcriptome of *D. magna-P. ramosa* interaction by exposing a host genotype to two parasite strains in a fully factorial design that included unexposed host controls. We sequenced transcriptomes at 4 h, 8 h and 12 h post exposure.

## Results

### Sequencing/mapping results

A total of 1,015,145,998 100 base pairs, paired-end sequencing reads were obtained. On average, 30 % of reads were identified as PCR duplicates and removed from further analyses. A total of 696,846,447 sequences were uniquely mapped over the 50 samples (mean 13,940,000 sequences/replicate, min = 11,180,000, max = 19,790,000).

### Infection profiles

The proportion of infected *D. magna* G4 hosts followed expected patterns based on previous infection studies [2, 8]. Specifically, *D. magna* G4 hosts exposed to *P. ramosa* infective S1 had a high proportion of individuals that became infected (N = 68/79, 86 %), whereas those that were exposed to *P. ramosa* non-infective S8 had no hosts that succumbed to infection (N = 0/67). No hosts that were exposed to the control solution of crushed *Daphnia* became infected (N = 0/72).

### Differentially expressed genes

#### Pairwise comparisons

**Fig. 1:**
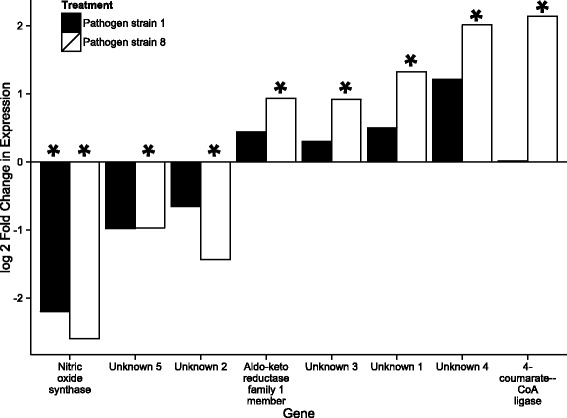
Log2 fold change in gene expression of *Daphnia magna* hosts exposed to *Pasteuria* S1 (an infective combination) compared to controls (black bars) and S8 (a non-infective combination) and controls (grey bars) four hours post exposure to the pathogen/control solution. Asterisks indicate that the pairwise comparison in the change in gene expression between pathogen treatment and control was significant at a FDR of 0.10. Functional annotation of the genes was carried out with BLASTp to the NCBI database

**Fig. 2:**
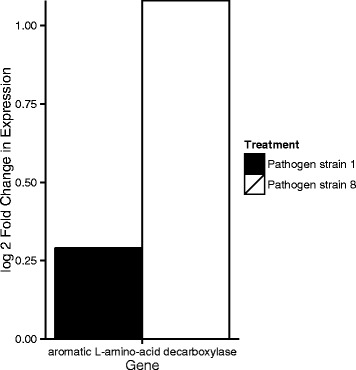
Log2 fold change in gene expression of *D. magna* hosts exposed to *P. ramosa* S1 (an infective combination) compared to controls (black bar) and S8 (a non-infective combination) and controls (grey bar) eight hours post exposure to the pathogen/control solution. The pairwise comparison in the change in gene expression between pathogen treatment and control was significant at a FDR of 0.10. Functional annotation of the genes was carried out with BLASTp to the NCBI database

Four hours post-exposure, one gene, inducible nitric oxide synthase (iNOS), was significantly downregulated in *D. magna* exposed to the infective spore (S1) compared to controls, and 8 genes were differentially expressed (5 upregulated and 3 downregulated) when exposed to the non-infective spore (S8), versus controls (Fig. 1). These eight genes included iNOS, a member of the aldo-keto reductase family, 4-coumarate—CoA ligase, and five genes whose sequences were not functionally characterized, and are hereafter referred to Unknown genes 1–5. Eight hours post-exposure, only an aromatic L-amino-acid decarboxylase gene was upregulated in *D. magna* exposed to S8 compared to controls (Fig. 2). In all other comparisons, no differentially expressed genes were identified.

#### GLMs

**Fig. 3:**
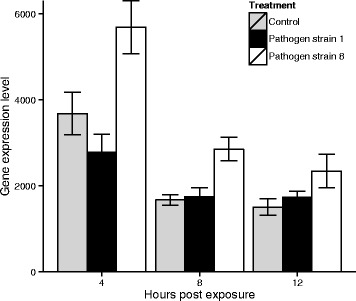
Expression values (in normalized mapped read count values) of a putative sulphotransferase (*D. magna* gene ID mu8AUG24b_p1s00687g88) identified as significantly different between the two treatments in the GLM (see methods). Data are shown for all three treatments (exposure to infecting *Pasteuria ramosa* S1, non-infecting S8 or a control solution) and three time points after treatment exposure (4, 8 and 12 h respectively). Error bars represent standard deviations

Only one gene, a putative sulfotransferase, was determined to be significantly differentially expressed in the test comparing the two-level factor ‘exposed’ and the three-level factor ‘treatment’ (p_adj_ = 0.02), indicating a potential difference between the effect of the infective and non-infective spore treatments. Visual inspection of the data suggests that this result is driven by an upregulation of this gene at all three time points in hosts that were treated with S8 (the non-infective spore) compared with hosts that were treated with S1 (the infective spore) or the control solution of crushed *D. magna* (Fig. 3). No differentially expressed genes were significantly associated with the interaction between time and treatment.

### Genome-wide patterns of transcriptional activity

**Fig. 4:**
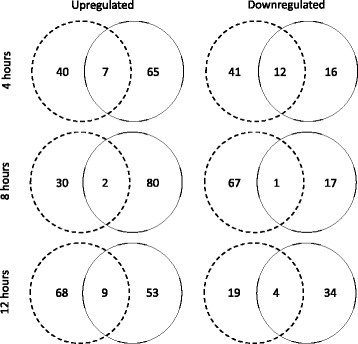
The proportion of up- versus downregulated genes in infective and non-infective treatments at 4,8 and 12 h post exposure. Genes were first ranked by uncorrected *p*-value, and the top 100 genes were selected. The first column displays the number of genes that are up-regulated. The second column displays the number of genes that are down-regulated. Dashed circles indicate the infective treatment, while solid circles indicate the non-infective treatment

The proportion of up- versus downregulated genes differed between the infective and non-infective treatments. At four and eight hours post pathogen exposure, the infective treatment showed a greater proportion of downregulated genes, while the non-infective treatment tended to upregulate genes (Fig. 4). At 12 h post pathogen exposure, the different treatments both have a higher proportion of upregulated genes. Furthermore, there was very little overlap in the genes that were either up or downregulated between the infective and non-infective treatments at any time point (Fig. 4).

### Quantitative PCR

**Fig. 5:**
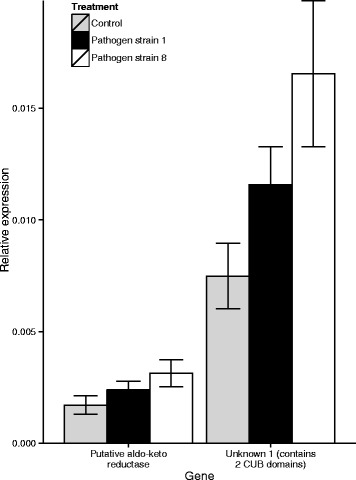
Quantitative PCR expression values of a putative aldo-keto reductase gene (*D. magna* gene ID mu8AUGep24bs00704g138) and Unknown gene 1 (containing 2 CUB domains) (*D. magna* gene ID mu8PASAgasmbl_16197) relative to actin (internal control) in three treatments at timepoint 4 h post exposure. Error bars represent standard deviations

We used qPCR to analyse the expression of two genes, unknown gene 1, which contains two CUB domains (*D. magna* gene ID mu8PASAgasmbl_16197) and aldo-keto reductase family 1 member (*D. magna* gene ID mu8AUGep24bs00704g138), in the samples that had been harvested four hours post pathogen exposure (Fig. 5). For both genes the pattern of gene expression exactly matched the pattern observed in the RNAseq data (Fig. 1): we found increasing expression from the control to S1 to S8. Furthermore, pairwise comparisons between the control and exposure to S8 (for which there was a significant difference in expression in the RNAseq data) yielded near significant results for Unknown gene 1 (*t*-test; d.f. =1,8; t = 2.52; *p* = 0.071). Results for the aldo-keto reductase family 1 member gene were not significant (*t*-test; d.f. =1,8; t = 1.94, *p* = 0.176), although the pattern was comparable to the RNAseq results.

## Discussion

### Differentially expressed genes

We identified eight genes that were differentially expressed (DE), compared to controls, four hours after pathogen exposure. Of these, only three had significant homology to functionally characterized genes from other arthropod species (inducible nitric oxide synthase (iNOS), an aldo-keto reductase family 1 member, and 4-coumerate-CoA ligase). iNOS has a clearly defined role in immunity, and has been documented to be upregulated after exposure to parasites in a number of taxa (e.g., [9–12]. Thus, it is unexpected to see *down*-regulation of this gene after pathogen exposure. Closer inspection of the data reveal that the down regulation appears to be driven by a spike in expression of the control treatment at timepoint 4, as the expression level of the gene appears to be unchanged in any of the treatments at any time point (Additional file 1: Figure S1). An absence of expression change is consistent with the results of Labbé et al. (2009) [13], who also found no change in expression of iNOS in *D. magna* clone G4 after exposure to *P. ramosa* S1, using qPCR. Unfortunately, our efforts to confirm our transcriptomic results with qPCR were not successful due to the overall low expression of the gene (i.e., average normalized read count = 149, versus 1221 for CUB and sushi domain-containing protein 1-like (mu8PASAgasmbl_16197) and 1091 for aldo-keto reductase family 1 member (mu8AUGep24bs00704g138)).

The biological function of the homolog of 4-coumarate CoA ligase gene in arthropods is unknown. This gene contains two highly supported domains, an AMP-binding and an AMP binding C-terminal domain. In bacteria, fungi, and plants 4-coumarate CoA ligase plays a metabolic role [14–16], however it is unclear if this is the case in other taxa. Whatever its function, in immunity or otherwise, our BLAST results indicate that the *D. magna* gene identified has high sequence identity to genes in other arthropods (e.g., 91 % sequence identity to the pea aphid), and thus it appears to be highly conserved. The last of the characterized genes encodes a member of the aldo-keto reductase superfamily, which consists of more than 40 enzymes and proteins. In mice, this protein is present in every tissue and as yet has no established link to immune function [17].

The remaining five genes all contained at least one characterized domain, which may provide some information about the protein’s putative function. For example, Unknown gene 5 (*D. magna* gene ID mPASAgasmbl62957) is a hypothetical protein that contains one copy of a chitin binding peritrophin domain. These domains are found in proteins that bind chitin, and in particular in chitinases of the peritrophic matrix. The peritrophic matrix lines the midgut of insects and crustaceans and, has been shown to filter out pathogens that may be ingested with food [18]. Given that the *D. magna-Pasteuria* route of infection may take place across the midgut, this protein warrants further study. Similarly, unknown protein 1 (*D. magna* gene ID m8PASAgasmbl_16197) contains two highly conserved CUB (complement subcomponents C1r/C1s, Uegf, Bmp1) domains. Proteins containing CUB domains can be involved in a diverse range of functions, including immunity. For example, in *C. elegans,* genes from the CUB-like gene family are upregulated after exposure to a bacterial pathogen [19].

The evidence for a direct role in immune function is weaker in the final three DE genes, however, this could simply be due to lack of suitable inquiry. Indeed two have the potential to play a role in host-pathogen interactions. Specifically, Unknown 2 (*D. magna gene* ID m8AUGep24b_p1s01361g121t1) contains 3 copies of an ankyrin repeat, a domain that modulates protein-protein interactions [20]. Unknown gene 4, (*D. magna gene* ID m8PASAgasmbl_45534), contains 6 leucine rich repeat motifs. This motif is involved in ligand recognition and signal transduction in the Toll-like receptor [21]. Finally, Unknown 3 (*D. magna gene* ID m8AUGepir7p2s01581g119t1) contains two partial domains, a fibrillar collagen C-terminal domain and C1q domain.

### Regulation and timing of transcriptional activity

Across the genome, it appears DE in resistant combinations tends towards up-regulation, whilst DE in susceptible combinations tends towards down-regulation of genes. This finding is consistent with a similar analysis conducted in bumblebees exposed to a trypanosome parasite [5]. The reason for the down-regulation is likely host immune suppression by the parasite, while the general up-regulation in non-infective *D. magna-P. ramosa* combinations is compatible with the idea that they are increasing the activity of defence genes. Pathogen interference with host gene expression has long been documented in other host systems. For example, the entomopathogenic bacterium *Xenorhabdus nematophila* has been shown to suppress expression of antimicrobial peptides in their host, the beet armyworm *Spodoptera exigua* [22]. Our data provide some evidence to suggest that a similar process might be taking place in this crustacean-bacterial host-pathogen system.

Intriguingly, this potential parasite interference with host expression matches the timing of host response. For example, we observed that host defence transcription occurs rapidly (i.e., four and eight hours post exposure, but not at 12 h). Parasite manipulation, should it prove to be occurring, also appears to happen immediately and then taper off (Fig. 4). It is hoped that the exploratory data presented here will stimulate further hypotheses, and further hypotheses testing centred both on host defence and parasite virulence strategies.

The difference in regulation between infected and non-infected individuals support the hypothesis that variation in resistance may be the result of variation in gene regulatory elements as well as, or instead of, variation in canonical immune system gene sequences. For example, it was found that house finches that were more resistant to a bacterium tended to upregulate immune system genes, whereas those that were susceptible tended to downregulate them [23]. Indeed, variation in gene regulation amongst host genotypes is well documented in insects (e.g., [24–26]), although the genes that are involved in such regulation are largely unknown. However, even given this limitation, Barribeau et al. (2014) [5] found a significant enrichment of gene ontology terms categories involved in regulating gene expression in bumblebees that were more susceptible to trypanosome genotypes. Our data suggest that variation in regulatory elements may also contribute significantly to the observed variation in resistance in crustaceans.

The infection process in the *D. magna-P. ramosa* system is generally thought to take place in two stages, namely, (i) pathogen entry into the host, governed by genetic compatibility between host and pathogen that is determined by proteins at the gut epithelial barrier and (ii) the innate immune response of the host upon pathogen entry. The relative contribution of genetic compatibility versus immunological response in determining host-pathogen specificity in the *Daphnia-Pasteuria* system is unclear. Dunneau et al. (2011) [6] found that *P. ramosa* spores attached to the *D. magna* oesophagus in genetically compatible (i.e., susceptible) host-pathogen combinations, but not in genetically resistant combinations. This suggests that genetic compatibility and not the immune response drives infection in this system. However, McTaggart, Wilson and Little (2012) [27] found that *D. magna* exposed to incompatible *P. ramosa* strains later had a decreased risk of infection when exposed to an infective strain. This suggests that genetically incompatible pathogen strains are detected and elicit a change in host’s immune response.

In support of the latter hypothesis, our transcriptome results suggest that it is largely in genetically incompatible, i.e., resistant, host-pathogen combinations where differential gene expression is observed (with the exception of iNOS, which is also differentially expressed in compatible combinations). Thus, it appears that some transcriptional regulation may play a role in preventing infection. Moreover, our results indicate that defence via gene regulation occurs very quickly after exposure, as DE genes were more readily detected early (at four hours), rather than later (at 12 h) in the experiment.

## Conclusions

The transcriptome profiles of *D. magna* exposed to an infective or a non-infective gram-positive bacterial strain has yielded several novel putative innate immune system genes that may play critical roles in the response to pathogens. The genome wide results indicate that the response to pathogen exposure is very rapid and transient, and that this timing profile may by mirrored by the pathogen’s response. Finally, as in other systems, it appears that transcriptional regulation may play an important role in determining the infection outcome of a particular host genotype.

## Methods

### Parasite exposures

This experiment used the naturally occurring host-parasite system *Daphnia magna* and *Pasteuria ramosa. Daphnia magna* are cyclically parthenogenic crustaceans that inhabit freshwater ponds. *Pasteuria ramosa* are spore-forming bacteria that cause sterilization, gigantism and premature death in *D. magna* [28]. Transmission of *P. ramosa* is exclusively horizontal, achieved by spores that are released from dead hosts and picked up by *D. magna* during filtration feeding [28].

We were interested in looking at transcriptional changes in response to parasite exposure, and how these changes varied over time and with parasite strain. We exposed a single *D. magna* host genotype (G4) to two parasite strains (S1, S8) or a control solution (C) over three time points post exposure (4 h, 8 h and 12 h) in a fully crossed exposure design. Past studies have shown that host genotype 4 is susceptible to S1 but resistant to S8 [2, 8], and this resistance pattern was confirmed in the present exposures (68/79 individuals infected when exposed to S1; 0/67 individuals infected when exposed to S2; 0/72 individuals infected when exposed to the control solution of crushed *D. magna*). Hereafter we refer to the use of S1 as ‘infective’, and the use of S8 as ‘non-infective‘.

Under favourable laboratory conditions, *D. magna* readily reproduces asexually, enabling genetic lines to be replicated for experimental purposes. Prior to the collection of individuals for the experiment, maternal stock lines were generated by placing groups of 20 female *D. magna* in jars (approximately 24) containing 200 ml of artificial culture medium. These maternal lines were grown over three generations in standard laboratory conditions (20 °C, 12 h light, fed 7x10^6^ cells of *Chlorella*/day) to remove potential co-variances due to maternal and grand-maternal effects. Individuals from the second clutch of the third generation maternal lines became the experimental animals (the first clutch was discarded).

Three hundred and sixty neonates from second clutches of the maternal lines were collected on the day of birth (all beakers were cleared of babies each day prior to collection) and males were discarded. These neonates were split into three exposure treatments (‘infective’, ‘non-infective’ or control). There were 6 replicates for each treatment each containing 20 individuals (so there were 120 individuals per treatment).

On day 5, *Daphnia* were exposed to pathogen spores in a 24-well cell culture plate. Due to space constraints within the well, each replicate of 20 individuals was divided into four groups and each group was placed in 1.5 ml of media in a single well, and exposed to 100 000 *P. ramosa* spores (S1 to make ‘infective’ combinations, S8 to make ‘non-infective’ combinations) or an equivalent volume of crushed uninfected *D. magna*. Spore numbers were estimated using a counting chamber (Neubauer-improved, 0.1 mm, Marienfield). All culture plates were then placed in an incubator at 20 °C, with light, for 4 h.

Immediately following pathogen exposure (time point four hours), the 20 individuals from each replicate were pooled back together. From each replicate (N = 6) within each treatment (N = 3), five individuals were removed and placed in single 1.5 ml tubes with 500 μl Trizol, ground with a pestle and stored at −80 °C for later RNA extraction. The remaining 15 individuals per replicate were placed in 200 ml beakers with fresh media and 7×10^6^ cells *Chlorella*, and maintained under standard laboratory conditions. Identical harvesting for RNA extraction was repeated at eight and 12 h post exposure. The final five individuals per replicate were maintained and visually monitored for infection until day 25. During the 25 day monitoring period, the media in the jar was changed twice weekly and *D. magna* were fed 5×10^6^ cells *Chlorella* daily.

### RNA extraction

Immediately before RNA extraction, an additional 500 μl of TRIzol (Ambion, Life Technologies) was added to each sample, and incubated for at least 5 min at room temperature. Two hundred μl of chloroform was added to each sample and shaken vigorously for 15–20 s. The samples were then centrifuged for 15 min at 4 °C at 11 600 rcf. The upper, aqueous phase was isolated and nucleic acids precipitated by adding 0.5 volumes of Absolute Ethanol, and inverting the tubes several times. This solution was used as the starting material for the RNeasy (Qiagen) protocol, which was followed according to the manufacturer’s instructions. After the last step of this procedure the isolated nucleic acids were subjected to a DNAse treatment. The integrity of the resultant total RNA was tested on Bioanalyzer (Agilent RNA-nano reagents). RNA and DNA concentrations were determined with a Qubit fluorometer (Invitrogen QuantRNA), while the 260:280 ratio was assessed on a Nanodrop (ThermoScientific).

### cDNA synthesis, library construction and sequencing

For each of the six replicates within each of the three treatments per time point, we subjected 5 μg of total RNA to one round of poly-A selection on oligo(dT) Serabeads (Illumina mRNAseq kits Cat no. RS-100-0801). The resultant messenger RNA was fragmented to an average size of 100 bp using divalent cations at 95 °C for 5 min prepared following the manufacturer’s recommended protocol (Illumina mRNAseq kits Cat no. RS-100-0801). First strand cDNA synthesis was carried out using Superscript III reverse transcriptase (Invitrogen) and 3 μg random hexamer primers (Illumina) per sample as per the manufactures’ instructions. Second strand cDNA synthesis and RNAseq samples were prepared according to the manufacturer’s recommended protocol (Illumina). The fragment size and concentration of resultant libraries were assessed on a Qubit fluorometer (Invitrogen QuantRNA) and on a Bioanalyser High Sensitivity Chip (Invitrogen QuantRNA). All libraries were sequenced on a GAIIX with 100 basepair, paired-end reads.

### Bioinformatic data preparation

We only retained reads that did not contain adapters, determined by a BLAST search. Furthermore, the fastx toolkit (http://hannonlab.cshl.edu/fastx_toolkit/) was used to only retain reads that had a Phred score greater than 20 over 75 % of the read length. These reads were aligned to the *D. magna* draft genome (v2.4) using TopHat 1.2.0 [29] and a .gtf file constructed from the most current version (Daphmagna_201104m8) of the gene models (N = 22164), which included the current data set as evidence. Default settings were utilized except for the following modifications: the minimum intron length was set to 50 nucleotides, the maximum intron length was set to 250 000 nucleotides, the minimum isoform fraction was set to 0.10, and only uniquely mapping reads were maintained (max-multihits was set to 1). Read pairs that had identical mapping coordinates were assumed to be the result of PCR error and flagged using the MarkDuplicate function in Picard (v 1.41), and not used in subsequent analyses. HTSeq-count (http://www-huber.embl.de/users/anders/HTSeq) was used to construct the count table using the default settings, except for mode, which was set to ‘intersection_nonempty’.

### Differentially expressed genes

Differential expression (DE) was determined using DESeq v2.13 [30]. We considered DE to be significant at a false discovery rate (FDR) of 10 %. In the first instance, we compared exposed *D. magna* hosts to control hosts to identify genes whose expression profiles changed after exposure to *P. ramosa*. This resulted in two pair-wise comparisons at each of the three experimental time points, for a total of 6 comparisons. Next, we used a GLM approach in DESeq v2.13 [30] to formally identify DE that was specific to the treatment (control, S1, S8). For this, we compared two models, the first included a two level factor that categorised hosts as either controls or exposed to the pathogen (regardless of strain). The second model included a three-level factor, pathogen S1 or S8 and control. We also tested for changes in gene expression over the experimental time course by testing if the interaction between treatment and time was significant. We tested between all models using a log-likelihood approach. All genes were functionally annotated with BLAST to the NCBI database, accepting the consensus annotation of hits scoring lower than 1e-05.

### Genome-wide patterns of transcriptional activity

In addition to identifying specific genes that were DE, we tested if the pattern of expression (i.e., up versus downregulated) differed between the two pathogen treatments in a large sample of potentially differentially expressed genes. Specifically, we compared the expression of the top 100 genes ranked by unadjusted *p*-value in the susceptible treatment to those in the resistant treatment. We conducted chi-square tests to determine, at each of the experimental time points, if the number of up and downregulated genes differed between the pathogen treatments.

### Validation of transcriptomic analysis using qPCR

We used comparative C_T_ (ΔΔC_T_) qPCR to confirm the expression of two genes (putative aldo-keto reductase: *D. magna* gene ID mu8AUGep24bs00704g138 and Unknown 1: *D. magna* gene ID mu8PASAgasmbl_16197). Using the same RNA samples that were prepared for RNASeq, complementary DNA (cDNA) synthesis was performed by mixing 250 ng total RNA with 250 ng random hexadeoxynucleotides (Promega) and heating to 75 °C for 10 min to denature RNA secondary structure. After chilling the samples on ice, 200 U MMLV reverse transcriptase (Promega), 5 μl 5x MMLV reverse transcriptase buffer, 1.25 μl dNTPs (Promega; final concentration 0.5 mM) and 20 U RNAsin RNase inhibitor (Promega) was added and the volume adjusted to 25 μl with nuclease free water. Samples were incubated at 37 °C for 60 min, followed by 70 °C for 15 min to deactivate the enzyme.

Real-time PCR was carried out using a StepOnePlus™ Real-Time PCR System (Applied Biosystems) and Fast SYBR Green Master Mix (Applied Biosystems) to monitor double-stranded DNA synthesis in combination with ROX as a passive reference dye. PCR reactions were carried out in duplicate using 7.5 *p*mol specific primers and approximately 5 ng cDNA in a total volume of 15 μl. The thermal profile for amplification was as follows: 95 °C for 2 min, followed by 40 cycles of 95 °C for 10 s, 58 °C for 30 s and 60 °C for 30 s. Primer pairs (sequences provided in Additional file 2: Table S1) were validated by standard curve analysis, and expression levels of target genes calculated using the ΔΔC_T_ method, with actin as the internal control gene. We designed primers with Primer3 (http://primer3.ut.ee/), with the exception of the actin primer pair, which were taken from [31]. We analysed the data using pairwise t-tests in which we tested for differences in expression between control animals and those exposed to S8 (the treatment for which there was a significant difference in expression in the RNAseq data).

## Availability of supporting data

All sequence data (raw Illumina reads) are available on the European Nucleotide Archive (ENA) under the accession ID ERP010925.

## Additional files

Additional file 1: Figure S1. Gene expression of iNOS at three timepoints after pathogen exposure. (PDF 97 kb) Additional file 2: Table S1. Quantitative PCR primer sequences. (DOCX 44 kb)

## Abbreviations

DE: Differential expression
cDNA: Complementary DNA
CUB: Complement subcomponents C1r/C1s, Uegf, Bmp1
iNOS: Inducible nitric oxide synthase
GLM: Generalized linear model
