# Supplementary figures and images for: Transcriptome profiling during a natural host-parasite interaction
Source: BMC Genomics. 2015 Aug 28;16(1):643. doi: 10.1186/s12864-015-1838-0 (PMC4551569; doi:10.1186/s12864-015-1838-0)

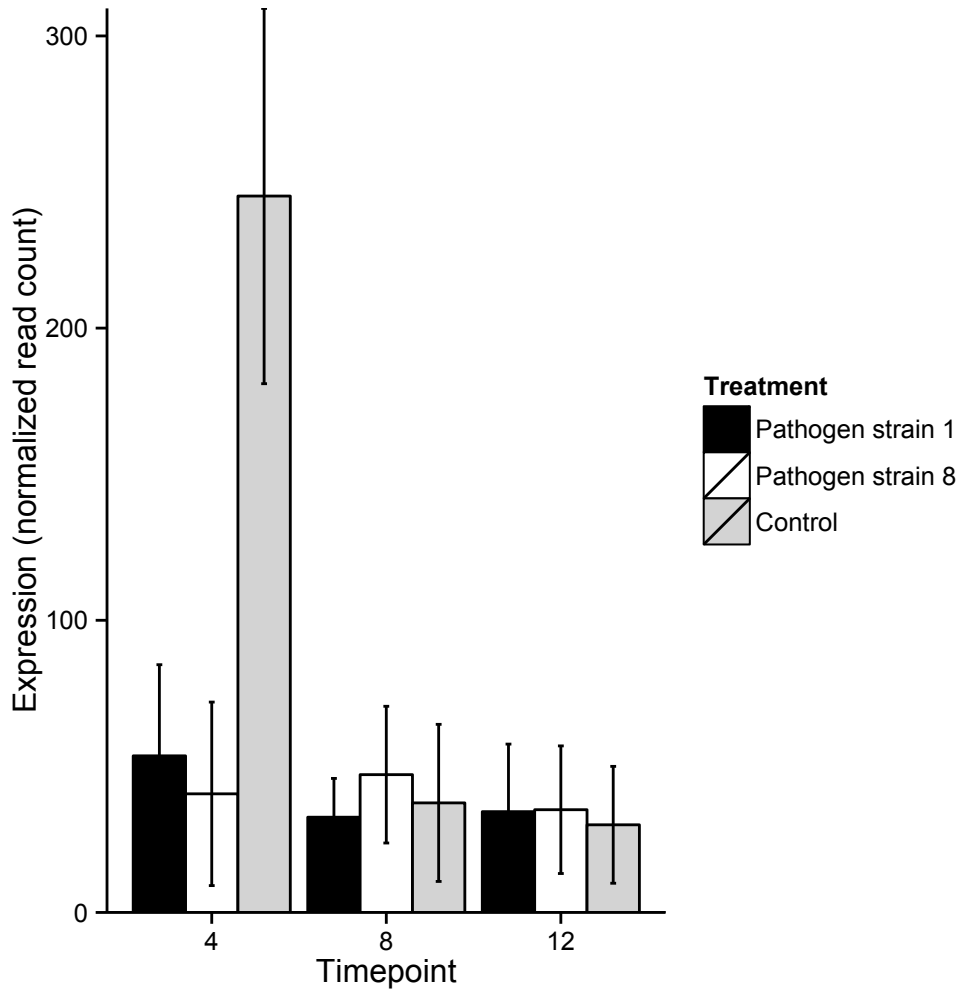

Supplement: Additional file 1: Figure S1. — Gene expression of iNOS at three timepoints after pathogen exposure. (PDF 97 kb) [file 12864_2015_1838_MOESM1_ESM.pdf]
