# Supplementary material for: Transcriptome profiling during a natural host-parasite interaction
Source: BMC Genomics. 2015 Aug 28;16(1):643. doi: 10.1186/s12864-015-1838-0 (PMC4551569; doi:10.1186/s12864-015-1838-0)
Supplement: Additional file 2: Table S1. — Quantitative PCR primer sequences. (DOCX 44 kb) [file 12864_2015_1838_MOESM2_ESM.docx]

Supplementary Table 1. Quantitative PCR primer sequences

| **Gene** | **F primer (5’ – 3’)** | **R primer (5’ – 3’)** |
| --- | --- | --- |
| Aldo-keto reductase family 1 member | TCG ACT AGA CTA CAT CGA CCT GT | AAC CGT CAG CAT TCA TGG GG |
| Unknown 1 | GCA TAG TTT TGT CCG CGT CG | AGC ACC AGT AAT CAA CCA GCA |
| Actin | CCA CAC TGT CCC CAT TTA TGA A | CGC GAC CAG CCA AAT CC |
